# A graph-based approach for the visualisation and analysis of bacterial pangenomes

**DOI:** 10.1186/s12859-022-04898-2

**Published:** 2022-10-08

**Authors:** Joshua D. Harling-Lee, Jamie Gorzynski, Gonzalo Yebra, Tim Angus, J. Ross Fitzgerald, Tom C. Freeman

**Affiliations:** 1 The Roslin Institute, Royal (Dick) School of Veterinary Studies, The University of Edinburgh, Easter Bush Campus, Edinburgh, EH25 9RG UK; 2 Roslin Innovation Centre, Easter Bush Campus, Edinburgh, EH25 9RG UK; 3 Janssen Immunology, 1400 McKean Road, Spring House, PA 19477 USA

**Keywords:** Bacteria, Pangenome, Accessory genes, Network graphs, Data visualisation

## Abstract

**Background:**

The advent of low cost, high throughput DNA sequencing has led to the availability of thousands of complete genome sequences for a wide variety of bacterial species. Examining and interpreting genetic variation on this scale represents a significant challenge to existing methods of data analysis and visualisation.

**Results:**

Starting with the output of standard pangenome analysis tools, we describe the generation and analysis of interactive, 3D network graphs to explore the structure of bacterial populations, the distribution of genes across a population, and the syntenic order in which those genes occur, in the new open-source network analysis platform, Graphia. Both the analysis and the visualisation are scalable to datasets of thousands of genome sequences.

**Conclusions:**

We anticipate that the approaches presented here will be of great utility to the microbial research community, allowing faster, more intuitive, and flexible interaction with pangenome datasets, thereby enhancing interpretation of these complex data.

**Supplementary Information:**

The online version contains supplementary material available at 10.1186/s12859-022-04898-2.

## Background

Early comparative genomic studies of bacteria revealed the extensive genetic variability between different isolates of the same species [1, 2]. As more genomes were sequenced and the true extent of genetic variation became apparent, the term ‘pangenome’ was coined to denote the full genetic repertoire of a group of isolates, typically defined at the species level [3]. Genes found in all, or nearly all, isolates are considered ‘core’ genes, while those found in some isolates but absent from others are termed ‘accessory’ genes. Depending on the species, more than 80% of the genes found in the species pangenome may be considered accessory [4]. Accessory genes frequently confer adaptive traits, including host/niche adaptation, virulence, and resistance to antimicrobials [5, 6]. The main drivers of accessory genome diversity are horizontal gene transfer mediated by mobile genetic elements, including phages, pathogenicity islands, and plasmids, along with recombination, gene duplication, and gene loss [7–12]. Consequently, the distribution of accessory genes across a population can be complex and difficult to unravel. Key questions remain unanswered, such as why some species have more diverse (‘open’) pangenomes than others, what the population-level drivers for differing gene frequencies are, and how to quantify the relative importance of accessory genes [4, 13–15].

Multiple computational tools have been developed to identify the full complement of genes from a given set of whole genome sequences, including Roary [16], PIRATE [17]**,** Panaroo [18] and PPanGGOLiN [19]. In all cases, the primary outputs constitute a core genome sequence alignment for phylogenetic analyses, a large presence/absence matrix for each accessory gene, and summary statistics and plots. Reconstruction of a pangenome is a computationally intensive task when the number of input sequences is large, and recent tools scale to the analysis of thousands of genomes. These tools also produce a graph (network) output file of the syntenic connections between genes across the pangenome. Whilst potentially useful, the resulting graphs are often very large and topologically complex, presenting a challenge to existing graph visualisation tools.

Effective visualisations allow a user to rapidly explore a dataset in a hypothesis-free manner. Furthermore, interactive visualisations provide real-time engagement with data, clear feedback on the effects of applied thresholds, and allow users to leverage their intuition and knowledge to investigations. Here, we discuss the application of the new 3D graph-analysis platform Graphia [20] to bacterial pangenome analysis, particularly as a visualisation strategy to allow interpretation of multiple aspects of bacterial pangenome datasets quickly, at scale, and on commonplace desktop hardware. We also provide scripts, collated in the GraPPLE repository (Graphical Processing for Pangenome-Linked Exploration), to aid the conversion of standard outputs from pangenome tools into appropriate formats for use in Graphia. Our approach is framed around the following questions:

1. Based on the accessory genome, how similar are a set of isolates to one another? How strongly associated are the observed groupings with the core genome phylogeny or other relevant categorisations, such as geographical location, habitat, clinical presentation, or sampling date?
2. How are accessory genes related based on their presence/absence across a collection of isolates? Are there significant associations between co-occurring gene clusters and isolate characteristics, e.g., the population clusters as defined in (1), the core phylogenetic groupings, or other known attributes?
3. What structural and syntenic relationships are there between core and accessory genes in the context of the full pangenome? Can useful information, such as function, be inferred from the position of a gene? Can such relationships inform us about the forces shaping genome evolution including the nature of mobile genetic elements and associated functions such as virulence or antimicrobial resistance?

## Results

**Fig. 1:**
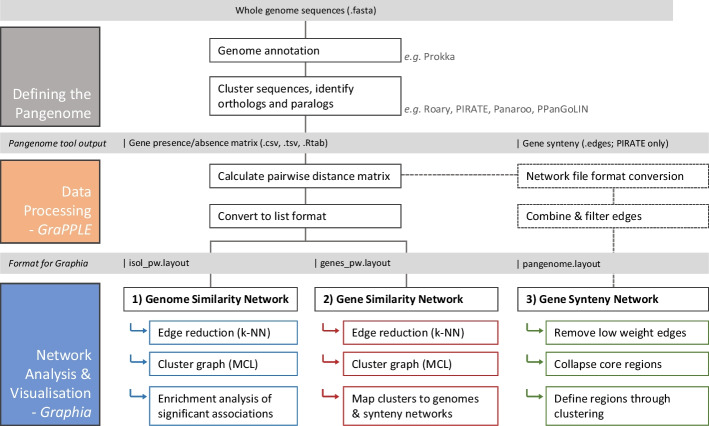
Overview of typical analysis workflow. Starting with whole genome sequences, the first step is genome annotation, then identification of elements of the pangenome, for which common tools are shown. The main input to the GraPPLE script library is the gene presence/absence matrix (in binary format). From this, pairwise Jaccard similarities are calculated, then converted to a list with annotations (compliant with the “.layout” file schema for Graphia load); as of version 3, Graphia can also load these matrices natively (see Additional file 1: Methods). Suggested filters and transformations to apply within Graphia are listed in order. GraPPLE also contains scripts to change the file formats of synteny graphs produced by common tools (where necessary) to allow for loading into Graphia*,* again with suggested transformations listed

From the output of a standard pangenome tool, it takes only minutes to run through the GraPPLE scripts and generate the three network files described. Loading and filtering within Graphia is similarly quick. A schematic summary is provided in Fig. 1. This approach is demonstrated below through case studies of two major pathogenic bacterial species: the highly clonal *Staphylococcus aureus* [21] and the highly recombinant *Legionella pneumophila* [22]. 778 *S. aureus* genomes from a previous study [23] and 379 *L. pneumophila* genomes from the NCBI database were used. All network files are available in the Additional file 3.

### Case study 1: *Staphylococcus aureus*

*Staphylococcus aureus* is a multi-host pathogen, responsible for disease burdens in humans and livestock: multiple lineages are able to switch host-species and adapt to cause disease in a new host [24, 25]. The mechanisms by which host switches occur often involve the acquisition of accessory genetic elements [23, 26]. Beginning with a previously assembled dataset [23], we demonstrate the use of network graphs to explore the population structure and accessory gene distribution within *S. aureus*.

#### Genome networks: overview of accessory genome similarity

The first type of graph represents the relationships between genome sequences based on the similarity of their accessory gene content. In these graphs, each node represents a single genome, and the edges between nodes are weighted by the Jaccard similarity coefficient (JSC) which is based on the number of shared genes over the total number of genes across the pair of isolates.

**Fig. 2:**
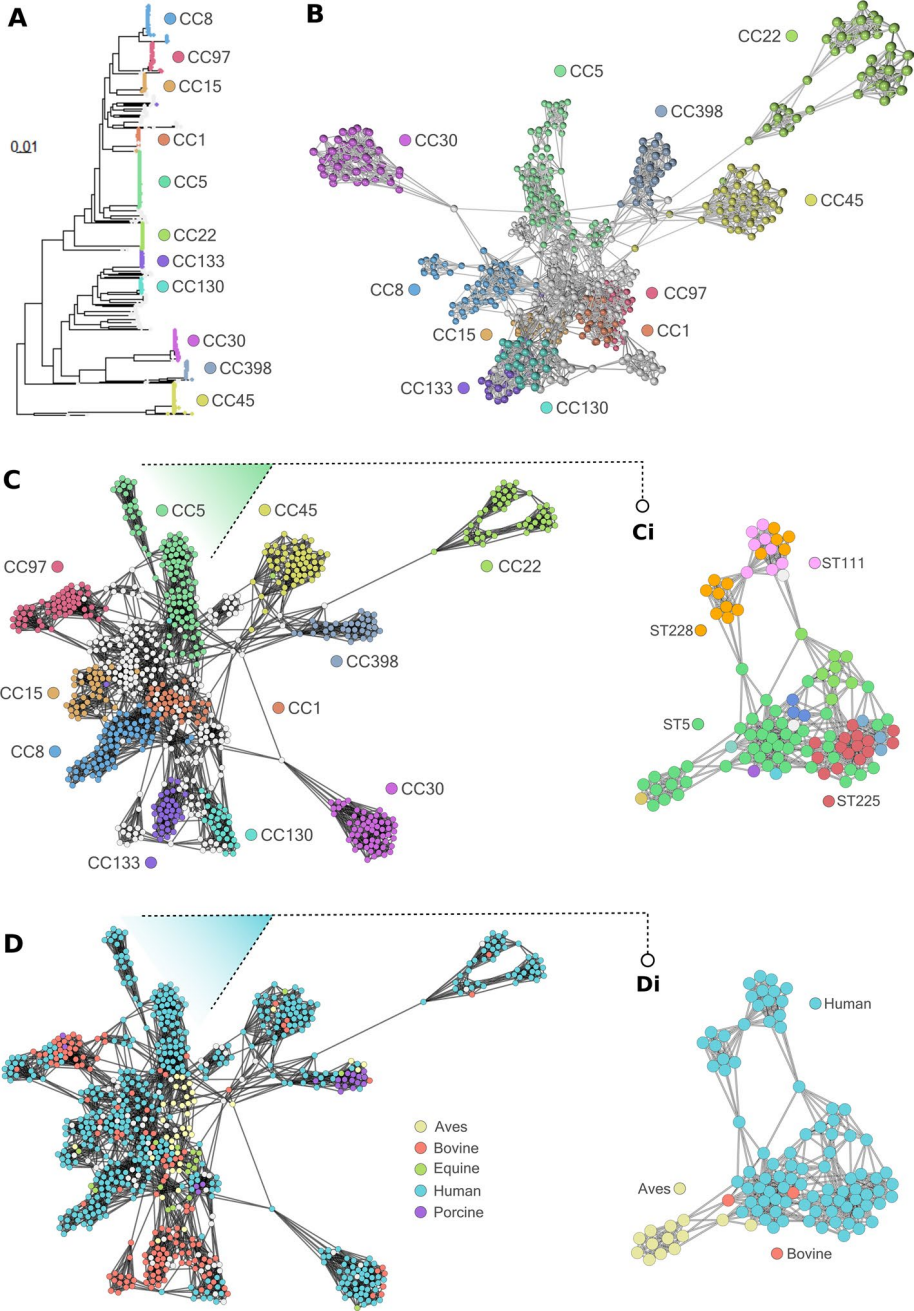
Genome-to-genome networks provide insight into population structure and associations. **A** Phylogenetic tree of all *S. aureus* isolates produced using ParSNP v1.2. Largest clonal complexes (CCs) are coloured and labelled, with minor CCs in white. **B** Relationship network between genomes based on the accessory genome visualised in 3D. Nodes represent individual isolates, edges represent shared accessory gene content (JSC > 0.8 filter applied), and a *k-*NN (*k* = 8) edge filter has been applied. The largest CCs are coloured and labelled, corresponding to the phylogeny in **A**. **C** The same network as in **B** but visualised using a 2D layout. **Ci** shows only CC5 isolates, demonstrating sub-structures within the network with nodes coloured by sequence type. **D** The same network as in **C** and **Di** the same network as in **Ci**, coloured by host

*Staphylococcus aureus* populations are highly clonal, with subtypes classified into sequence types (STs) and broader clonal complexes (CC) based on the allelic profile of seven core genes. These groups are highly consistent with core genome phylogeny (Fig. 2A). After transformations were applied (see Methods), the resulting genome-genome similarity graph comprised 778 nodes and 4483 edges. The relationship between core phylogeny and accessory genes can be visualised by colouring each genome according to the CC to which it belongs. We observed strong visual correlation between network structure and CC (Fig. 2B, C), and between network structure and ST within individual CCs (Fig. 2Ci). Formal statistical testing of such associations is supported by Graphia through the built-in Enrichment Analysis tool, which calculates an adjusted Fisher’s p-value between each pair of values across two attributes, typically network clusters against a metadata variable. Here, the association between CC and Markov Cluster (MCL) clusters (inflation value (MCLi) = 2.00) were tested. 44/45 MCL clusters were significantly associated with at least one CC (adj. *p* < 0.05). This close relationship between core and accessory variation within each lineage is consistent with known lineage restriction barriers that limit horizontal gene transfer between CCs in *S. aureus* [21].

Colouring the genome network by host species visually distinguishes groupings of similar genomes linked to single or multiple host-species (Fig. 2D). The network can also be interactively filtered on node attributes to investigate a subset in more detail. For example, retaining only genomes from CC5 shows a clear separation between genomes from human and avian hosts in this clade (Fig. 2Di).

These genome graphs are a useful method for visualising the global relationships between all isolates simultaneously, reflecting the complex and “non-vertical” patterns of accessory genome content. The JSC gives the direct proportion of shared accessory genes between each pair of genomes and is thus the preferred (default) metric used. Other pairwise measures, such as Euclidean and cosine distances, are supported in the GraPPLE scripts.

#### Gene association networks: identifying shared sets of accessory genes

The second graph type is a gene co-occurrence network, calculated from the same matrix as the genome network, but inverted. In this instance nodes represent genes, and edges are weighted (JSC) between each pair of genes based on co-occurrence across the population. Clustering the graphs defines groups (sets) of genes with highly similar prevalence.

**Fig. 3:**
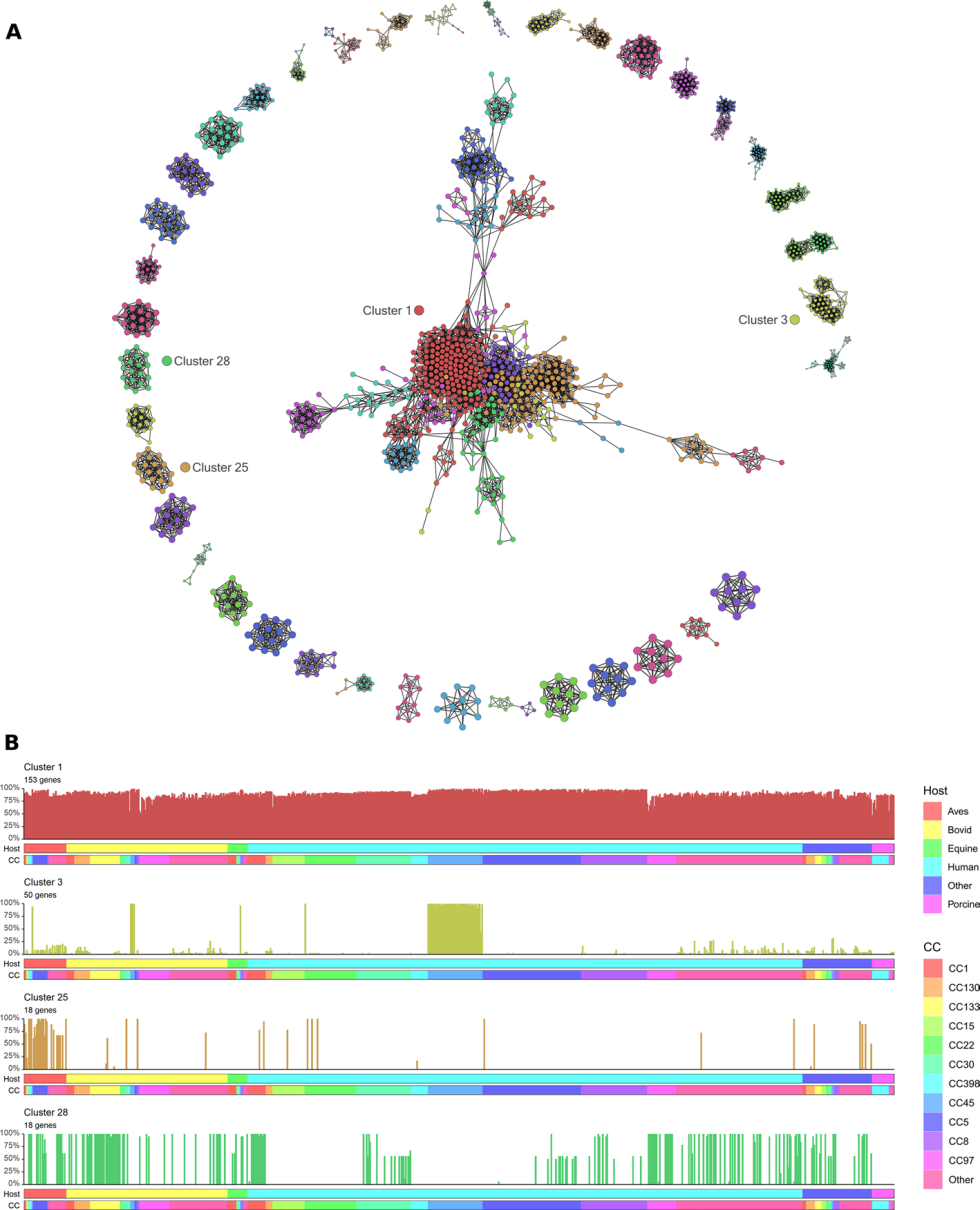
Gene association networks provide relationship between gene presence and host genome characteristics. **A** A filtered gene-gene association graph visualised in 2D space. Each node represents a gene and weighted edges correspond to the Jaccard similarity coefficient between each pair of genes (JSC > 0.550). High and low frequency genes have been removed (n < 7; n > 770), as have smaller components (n < 10), for visual clarity. Nodes are coloured by cluster (MCLi = 1.50). **B** Four gene distribution profile plots, representing commonly observed distributions: Cluster 1, near-core genes; Cluster 3, set of genes showing a near lineage-specific distribution (CC45); Cluster 25, host-associated (aves) set of genes; Cluster 28, widely distributed but not lineage-specific set of genes. Each bar in a plot represents an individual genome, with bar height equal to the proportion of genes in the cluster present in that genome. Coloured horizontal bars below the x-axis represent the host (upper) and clonal complex (lower)

After applying transformations (see Methods), the gene–gene similarity network comprised of 1926 nodes and 8769 edges across 285 components (groups of connected nodes). The genes were clustered (MCLi = 1.50), and clusters ranged in size from 2 to 146 genes (Fig. 3A). Clusters are ordered by size, in descending order, and four gene clusters with characteristic profiles are shown in Fig. 3B. Cluster 1, the largest cluster, contained 153 genes that are present in the majority of isolates. Cluster 3 contained 50 genes specific to CC398; many other clusters are similarly lineage-related or restricted, consistent with the strong lineage signal observed in the genome-genome similarity networks. Cluster 25 contained 18 genes with high occurrence in genomes from strains found in the avian host, consistent with divisions seen in the genome-genome graph. Cluster 28 contained 18 genes present in a range of isolates, implying a high gain/loss rate, and a high number of these genes contain phage-associated annotations.

#### Gene synteny networks: the pangenome visualised as a 3D graph

The third graph type represents the syntenic relationships between genes. In such a network, nodes represent genes and edges are weighted according to the number of times any two genes are observed next to each other across the population. The large size and complex topologies of these networks mean they are challenging to render and interpret. Compared to existing graph tools recommended for the visualisation of pangenome graphs, such as Cytoscape [27], Gephi [28], and Bandage [29], Graphia improves the topological representation of the networks through a 3D layout, and increases interactivity with real-time layout and application of thresholds (Additional file 1: Fig. S1).

The primary *S. aureus* synteny graph comprised of 7091 nodes, connected by 17,100 edges (Additional file 1: Fig. S2A). The first transformation removed ~ 6000 low frequency edges, chosen here as those with weight < 8 (< 1% of isolates; Additional file 1: Fig. S2B). In these examples, nodes are also removed when the removal of an edge disconnects any node(s) from the largest graph component. These nodes are almost exclusively low frequency genes, the majority of which were annotated as “hypothetical”. A second transformation was applied, contracting all edges which occur in over 99% of genome sequences (weight > 770). Where there are stretches of highly conserved syntenic genes, this transformation collapses these regions down to a single node (Additional file 1: Fig. S2C). This mirrors the removal of such “near” core genes from the gene association networks but retains the contextual information as links between variable regions. Further` low frequency genes were also removed (*n* < 10). The resulting network comprised of 2711 nodes and 4110 edges. The node with the highest multiplicity represented 29 genes, the majority of which are predicted to encode 30S and 50S ribosomal subunits. To broadly classify regions of the pangenome, and aid subsequent filtering, the Louvain clustering (LC) algorithm was used to cluster the network (inflation value = 0.400), resulting in 18 clusters (Additional file 1: Fig. S2D).

**Fig. 4:**
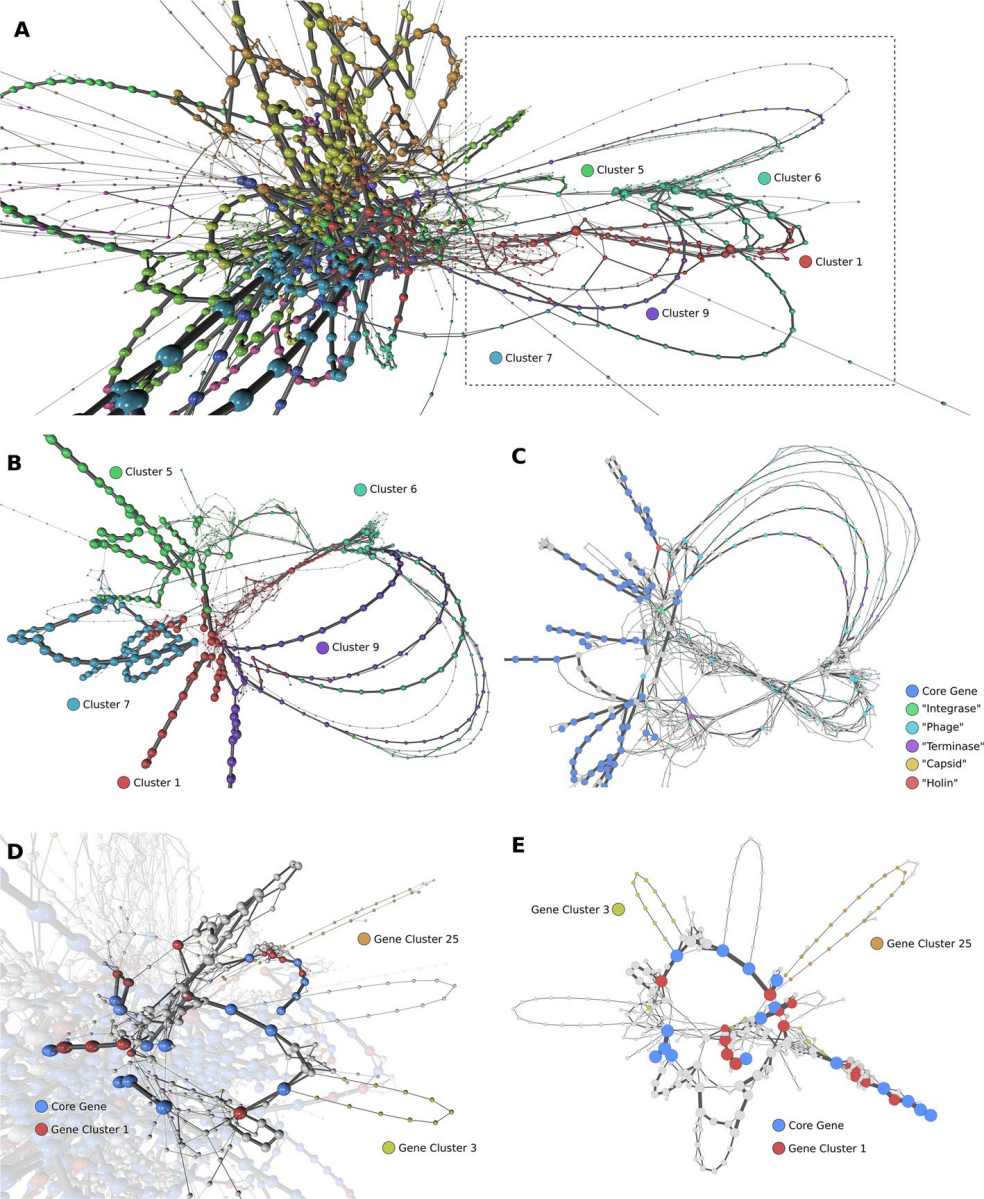
Syntenic connections within the *S. aureus* pangenome shows location of gene sets. **A** Full synteny network of *S. aureus* at 90% gene identity. Nodes represent genes and are sized according to the number of isolates in which they occur. Edges are weighted as the number of times two genes occur next to one another. Coloured by Louvain Cluster (LCi = 0.400). **B** “Phage” region (right-hand side of network in **A**), selected by retaining only nodes in clusters 1, 5, 6, 7 and 9. Coloured as in **A**. **C** 2D layout of the same region as in **B**, with common annotation highlighted alongside core genes. This network allows detailed inspection of phage integration sites with respect to core genes, accessory genes shared between common phage types, and putative novel factors carried on phage variants. Gene cluster 28 (see Fig. 3) is also found in this region. **D** Region, in context, that carries gene cluster 25 (orange; see Fig. 3) as a single syntenic set, inserted next to a near-core gene. Examples of gene cluster 3 (yellow) sets are also seen. **E** 2D layout of region from **D** (clusters 2 and 18). Smaller, focused networks aid navigation and investigation of specific gene locations and neighbours

These networks capture population-wide variation in specific regions, thereby allowing visual identification of regions of interest (Fig. 4A). For example, we observe an area of higher variation in the *S. aureus* synteny network (Fig. 4A, dotted box). Filtering to only the LCs found in this area, we note a high proportion of “phage” annotations (Fig. 4B). This “phage” region can be resolved to multiple different paths, each representing different arrangements of integrated phage gene sets across the population; annotations give further context to these regions (Fig. 4C).

Mapping clustering information from the gene–gene similarity graphs to the synteny network provides a visual schematic of which genes are shared across which genomes. For example, we searched for genes from cluster 25 (Fig. 3B; orange) and highlighted the region (defined by Louvain cluster) in which they occur (Fig. 4D). Filtering the network to this region shows gene cluster 25 occurs as a single syntenic set of genes, connected to a putative lipoprotein gene (red; Cluster 1). Two small sets of genes from gene cluster 3 (yellow) are also present in this region; other genes from gene cluster 3 are observed as small sets of genes distributed across the pangenome graph. These have likely diverged from common ancestors since the lineage emerged or are genes that have become fixed in this lineage after acquisition events. Genes from gene cluster 28 were found in a single syntenic block in the prophage region shown in Fig. 4C; the known higher mobility of prophage genes such as these is consistent with the sporadic distribution seen in the profile of this gene cluster (Fig. 3B).

### Case study 2: *Legionella pneumophila*

*Legionella pneumophila* is a globally ubiquitous, freshwater species which causes Legionnaires’ disease, a severe form of pneumonia [30, 31]. *L. pneumophila* is an opportunistic human pathogen and most infections are sporadic. However, outbreaks have been linked to a variety of environmental reservoirs, which pose a major public health threat [32]. Here, we used the methods described above to investigate the pangenome of *L. pneumophila* using a dataset of 379 genome assemblies from the NCBI database, selected to represent the known breadth of species diversity.

**Fig. 5:**
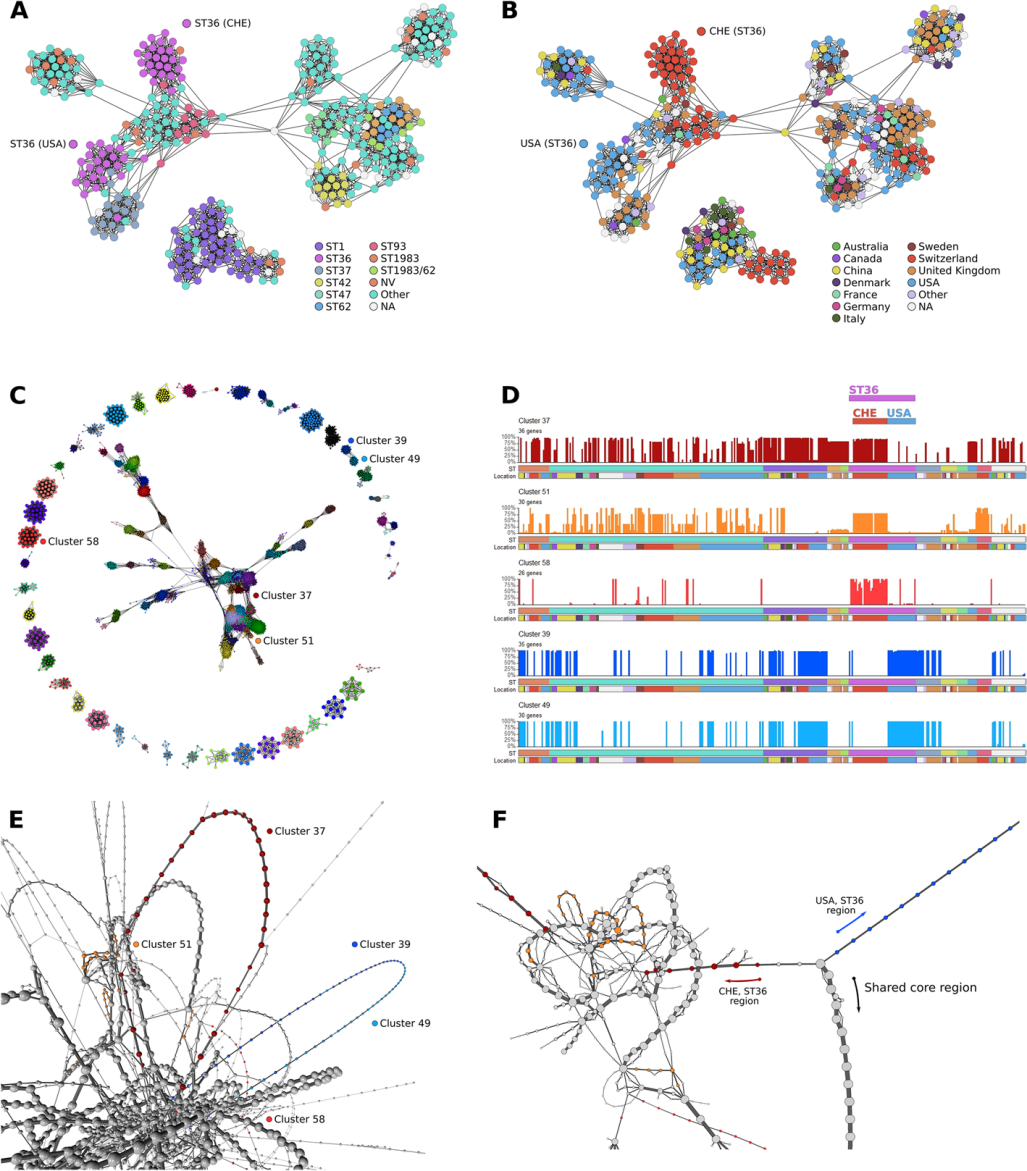
Investigating the *L. pneumophila* pangenome through network graphs. **A** Genome-genome similarity network, coloured by ST. Nodes represent genomes, and edges are weighted by pairwise JSC. Two key ST36 clades are highlighted. Edges are filtered by JSC > 0.5 and using a k-NN = 10. **B** Same network as in **A** but coloured by sampling location. Same two key ST36 clades are highlighted. **C** Gene–gene similarity network; nodes represent genes, edges weighted by pairwise JSC. Edges are filtered by JSC > 0.55, and k-NN = 20. Clustering with MCLi = 1.50. Components of *n* < 10 removed for visual clarity. **D** Accessory gene distribution plots of the largest gene clusters associated with the division in ST36 between Swiss (reds and orange) and US (blues) genomes. Plots as described in Fig. 3. **E** 3D rendering of the pangenome synteny graph for *L. pneumophila*; coloured nodes correspond to the clusters in **C**, **D**. **F** Filtered and 2D view of the key region, with convergence of both Swiss and US-associated regions to the same core region

The *L. pneumophila* pangenome was produced using PIRATE at the 90% identity threshold, constituting 2029 core genes (present in > 99% isolates) and 8456 accessory genes. Pairwise JSCs between genomes and genes were calculated as described above. The genome network contained 379 nodes and 2693 edges, in 12 clusters (MCLi = 1.40). Through comparison of ST (Fig. 5A) and location (Fig. 5B), we can identify two distinct clusters of ST36 genomes associated with a geographical division (US and Switzerland).

To detect the difference in gene content causing this division in ST36, we use the gene–gene similarity network. After filtering (see Methods), this network consisted of 4270 genes and 49,000 edges, which formed 201 clusters (MCLi = 1.50; Fig. 5C). The distribution profiles of the five largest gene clusters associated with the ST36 division are shown in Fig. 5D. Three of these clusters were identified in Swiss ST36 genomes (red and orange; Fig. 5C, D), whilst two clusters were found in US ST36 genomes (blue; Fig. 5C, D). The two US-associated clusters were from the same component of the gene network, consistent with the high similarity of their distribution plots. These clusters were then mapped onto the synteny graph, and each were found to be in syntenic, connected blocks across the population (Fig. 5E). One end of each of these blocks converged on the same core region (Fig. 5F), implying either an insertion/deletion event mediated by a mobile genetic element, or a recombination event. Further investigation of this region in the Philadelphia-1 reference genome (Genbank Accession: ASM8485.1) showed one side to be flanked by coding sequences encoding tRNA, as well as an integrase (*intD*), suggesting that the region may be part of an integrative and conjugative element [33]. The other side of the region was flanked by genes annotated as transposases and contained additional genes with annotations suggesting a role in conjugation, providing further evidence of mobility; this is consistent with the sporadic presence/absence of the genes across the rest of the *L. pneumophila* population.

## Discussion

The complexity of accessory gene distribution presents a challenge to finding meaningful associations between gene sets, population structure, and phenotype. The first type of network graph presented in this study is comparable to other visual representations of the population structure based on accessory genes, including those produced by PANINI [34] and PopPUNK [35]. PANINI utilises t-distributed stochastic neighbour embedding (t-SNE) to plot isolate relatedness, representing the similarity of accessory genomes in 2D space, while PopPUNK calculates the distance between core and accessory regions for all pairs of isolates through k-mer comparison. Both are useful summary visuals and groupings, but the 3D and interactive approach demonstrated here enables greater exploration of the underlying data structure, and perception of the impact applied thresholds have on that structure.

The simple method of grouping genes based on JSC defines sets of genes with similar prevalence across bacterial populations. These clusters help contextualise further investigation within synteny networks, as presented here, and in other tools. For example, genome-wide association studies, which test the significance of associations between gene presence and particular traits such as virulence or host adaptation [36]; Pantagruel [37], which describes co-evolving gene sets by reconciling gene trees to the species tree; or Coinfinder [38], which identifies genes that share common or opposing patterns of inheritance or loss across the population.

Regarding synteny graphs, recent tools have sought to utilise these further: Panakeia [39] systematically quantifies the structures seen within these graphs, while MetaPGN [40] provides a schematic representation, though scalability remains a limitation with both. Cytoscape, Gephi and Bandage have restricted, 2D layout options, and the complexity of the networks can lead to frame rate and graphics rendering challenges. Graphia addresses many of the limitations of current network tools and greatly increases the usability of these data. Enabling visualisation and investigation of these networks in full, in 3D, and with clustering context for accessory genes, will allow for insights into gene sets not previously investigated and those of “hypothetical” function. We envisage this approach being particularly effective in less commonly studied species, speeding up investigation of accessory genetic elements and subsequent analyses.

Finally, the case study presented in *L. pneumophila* demonstrates the application of these network graphs in tandem to: (1) identify divisions in a population that reflect variation in accessory gene content; (2) identify the specific genes associated with that division, and; (3) establish the syntenic context of those genes through the power of visualisation. Taken together, the analysis led to the identification of a putative novel mobile genetic element associated with geographic division in *L. pneumophila* ST36, one of a limited number of important global pathogenic clones [41].

## Conclusion

Modern sequencing platforms are now generating vast amounts of data describing the genomic diversity within and across bacterial species. However, the sheer size and complexity of such datasets presents major challenges for existing tools, particularly with respect to visualisation and interpretation.

The pangenome tools Panaroo, PIRATE and PPanGGOLiN all produce gene count matrices which, as demonstrated here, can be used to generate graphs for analysis purposes. These tools also produce graphical formats of pangenome synteny. Graphia has numerous advantages when working with such data but requires the data to be formatted appropriately; the GraPPLE repository was initially developed to address this, though functionality is also being added directly to Graphia.

Our approach renders highly informative visual representations of the complex relationships within the bacterial pangenome at a level of detail and speed not previously possible, especially with large datasets. We anticipate that the approach and the resources described here will be applied to understand the adaptive evolutionary biology of a wide variety of important bacterial species.

## Methods

### Dataset preparation

*S. aureus* genome assemblies were taken from a previous study [23]. *L. pneumophila* assemblies were downloaded from the NCBI database (18/02/2020), and down-sampled using Assembly-Dereplicator (github.com/rrwick/Assembly-Dereplicator). Metadata were downloaded from the relevant online repositories. The sequence types of *S. aureus* isolates were determined through reference to PubMLST (pubmlst.org) using mlst (github.com/tseemann/mlst). ParSNP v1.12 [42] was used to produce a core gene alignment and generate a phylogenetic tree, visualised using ggtree [43]. *L. pneumophila* ST was determined using chewBBACA [44]. All analyses up to and including the GraPPLE scripts were run on a standard Cloud Infrastructure for Microbial Bioinformatics (CLIMB) [45] virtual machine.

### Genome annotation and pangenome definition

All assemblies in FASTA format were annotated using Prokka v1.14.6 [46] with default databases, specifying the appropriate genus. The annotated files in GFF3 format were used as input to PIRATE v1.0.4, and the pangenome for each species determined under default settings. The resulting presence/absence matrix was filtered to only genes at the 90% id threshold.

### Genome-genome graphs

The initial graph of *S. aureus* genomes consisted of 778 nodes and 302,253 edges. Applying first an edge threshold of JSC > 0.8 within Graphia reduced the edge count to 265,400, and then the k-Nearest Neighbours (k-NN) algorithm (k = 8) reduced the edge count further to 4483. Clustering (MCLi = 2.00) resulted in 45 clusters. An adjusted Fisher’s p-value between MCL cluster and CC was calculated using the built-in Enrichment Analysis tool in Graphia. In the *L. pneumophila* analysis, the raw network consisted of 379 nodes connected by 70,100 edges; following the application of k-NN (k = 10) the edge count was reduced to 2693, forming 2 components. Lower weight edges were kept here to better retain relational connectivity. The graph was clustered with MCLi = 2.00, resulting in 15 clusters.

### Gene–gene graphs

The initial graph of *S. aureus* genes contained 5368 nodes (genes) and 2.7 million edges, across 299 components. Genes were removed if they were found in > 99% genomes (removing 2015 genes) or < 1% genomes (removing 1307 genes), and edges of JSC weight < 0.55 were also removed. These transformations reduced the networks to 2064 nodes and 41,700 edges across 314 components. The k-NN algorithm (k = 10) was used to reduce edge density further to 9196. The resulting graph was clustered at MCLi = 1.50, giving 264 clusters and 95 single node components. The initial *L. pneumophila* gene–gene similarity graph contained 6702 nodes and 1.5 M edges, across 118 components. Genes were removed if they were found in > 99% genomes (removing 910 genes) or < 1% genomes (removing 1522 genes), and edges of JSC weight < 0.55 were also removed. The k-NN algorithm (k = 20) removed a further 267,700 edges. The resulting graph of 194 components was clustered at MCLi = 1.50, giving 201 clusters. Gene cluster profile plots were produced using the “plot_gene_cluster_profiles.R” script.

### Synteny graphs

Synteny graphs were created for genes at the 90% id threshold by running the “pangenome_graph.pl” script from PIRATE, with a modified gene presence/absence matrix at 90% as input (moving alleles to gene families using the “generate_edges.sh” script from the GraPPLE repository). The synteny file was converted to the “.layout” format using the “py_edges_to_layout.py” script from the GraPPLE repository with default settings. *S. aureus* transformations are described in the Results section to demonstrate simplification. The *L. pneumophila* graph was produced similarly through recreation of the “.edges” file with PIRATE adapter scripts, at the 90% id threshold. This file was converted to “.layout” using GraPPLE script “edges_to_layout.py”, loaded to Graphia, and simplified by removing edges of weight < 10, and contracting edges of weight > 370.

### Network transformations

All network transformations listed above were carried out using the Graphia user interface unless specified. For more information on specific transformations see the Graphia User Guide (graphia.app/userguide.html). Graphia (v2.2) analysis performed on a standard-specification laptop (Intel Core i7-7500U @ 2.70 GHz, 8 GB RAM, integrated Intel HD Graphics 620, Windows 10 Pro).

## Supplementary Information

**Additional file 1. **Supplementary Methods and Figures.

**Additional file 2: Table S1.** Metadata for the *Legionella* genomes used in the study.

**Additional file 3: Data 1.** Graphia file for the *S. aureus* genome-genome similarity network, as in Figure 2. **Data 2.** Graphia file for the *S. aureus* gene-gene similarity network, as in Figure 3. **Data 3.** Graphia file for the *S. aureus* gene synteny network, as in Figure 4. **Data 4.** Graphia file for the *L. pneumophila* genome-genome similarity network, as in Figure 5. **Data 5.** Graphia file for the *L. pneumophila* gene-gene similarity network, as in Figure 5. **Data 6.** Graphia file for the *L. pneumophila* gene synteny network, as in Figure 5.

## Data Availability

All sequences and metadata used in this paper are available from public repositories; a list is provided in the Additional file 2. Graphia is a free, open-source software available from graphia.app under a GNU General Public License v3.0. GraPPLE scripts are available on GitHub (JDHarlingLee/GraPPLE).

## Abbreviations

CC: Clonal complex
JSC: Jaccard similarity co-efficient
k-NN: k-nearest neighbours
LC: Louvain Cluster
MCL: Markov Cluster
MCLi: Markov Cluster inflation value
ST: Sequence type
t-SNE: t-distributed stochastic neighbour embedding
