# Supplementary material for: A graph-based approach for the visualisation and analysis of bacterial pangenomes
Source: BMC Bioinformatics. 2022 Oct 8;23:416. doi: 10.1186/s12859-022-04898-2 (PMC9548110; doi:10.1186/s12859-022-04898-2)
Supplement: Supplementary file 1 — Additional file 1. Supplementary Methods and Figures. [file 12859_2022_4898_MOESM1_ESM.docx]

**Supplementary Information**

**A graph-based approach for the visualisation and analysis of bacterial pangenomes**

Joshua D. Harling-Lee^1*^, Gonzalo Yebra^1^, James Gorzynski^1^, Tim Angus^2^, J. Ross Fitzgerald^1^ and Tom C. Freeman^1,2,3*^

1. The Roslin Institute, Royal (Dick) School of Veterinary Studies, Easter Bush Campus, The University of Edinburgh, EH25 9RG.

2. Roslin Innovation Centre, Easter Bush Campus, Edinburgh, EH25 9RG.

3. Janssen Immunology, 1400 McKean Road, Spring House, PA 19477 US.

*Corresponding Authors: [j.d.harling-lee@roslin.ed.ac.uk](mailto:j.d.harling-lee@roslin.ed.ac.uk), [tfreema6@its.jnj.com](mailto:tfreema6@its.jnj.com)

**Supplementary Table 1.** Metadata for all *L. pneumophila* isolates used in Case Study 2

**Supplementary Data 1.** Graphia file for the *S. aureus* genome-genome similarity network, as in Figure 2.

**Supplementary Data 2.** Graphia file for the *S. aureus* gene-gene similarity network, as in Figure 3.

**Supplementary Data 3.** Graphia file for the *S. aureus* gene synteny network, as in Figure 4.

**Supplementary Data 4.** Graphia file for the *L. pneumophila* genome-genome similarity network, as in Figure 5.

**Supplementary Data 5.** Graphia file for the *L. pneumophila* gene-gene similarity network, as in Figure 5.

**Supplementary Data 6.** Graphia file for the *L. pneumophila* gene synteny network, as in Figure 5.

## **Supplementary Methods**

## **GraPPLE Pipeline Overview**

The GraPPLE repository supports the generation of three graph types to address the three questions above, as outlined in Figure 1: (1) the clustering of genomes based on accessory gene content; (2) the clustering of genes based on their shared occurrence across the population; and (3) the visualisation of syntenic relationships (the order in which genes occur).

The main GraPPLE scripts are written in python v3.6 and are available from GitHub (github.com/JDHarlingLee/GraPPLE). The primary pairwise script requires the sklearn.metrics, numpy and pandas modules. Gene presence/absence matrices were converted to binary (.tsv) format using the PIRATE adapter script ‘PIRATE_to_Rtab.pl’. The pairwise Jaccard similarity coefficient (JSC) was calculated in each instance from the resulting binary matrices using the ‘pw_similarity.py’ script, with default settings.

**Comparison of Synteny Graph Rendering**

Cytoscape (v3.9.1), Gephi (v0.9.5) and Graphia (v3.0) were compared in their loading, layout and interactivity. The pangenome synteny network from the *S. aureus* dataset (as in Figure 4) was loaded to Graphia and two files saved in .graphml format – a full network, with all nodes and edges, and a filtered network with genes in only more than 10 or less than 770 genomes. Force directed layouts with default settings were used in each program (Force Atlas Layout 2 in Gephi, OpenCL Prefuse Layout algorithm in Cytoscape and the native force directed layout in Graphia). A 3D layout was attempted in Cytoscape using the Cy3D plugin but was unsuccessful.


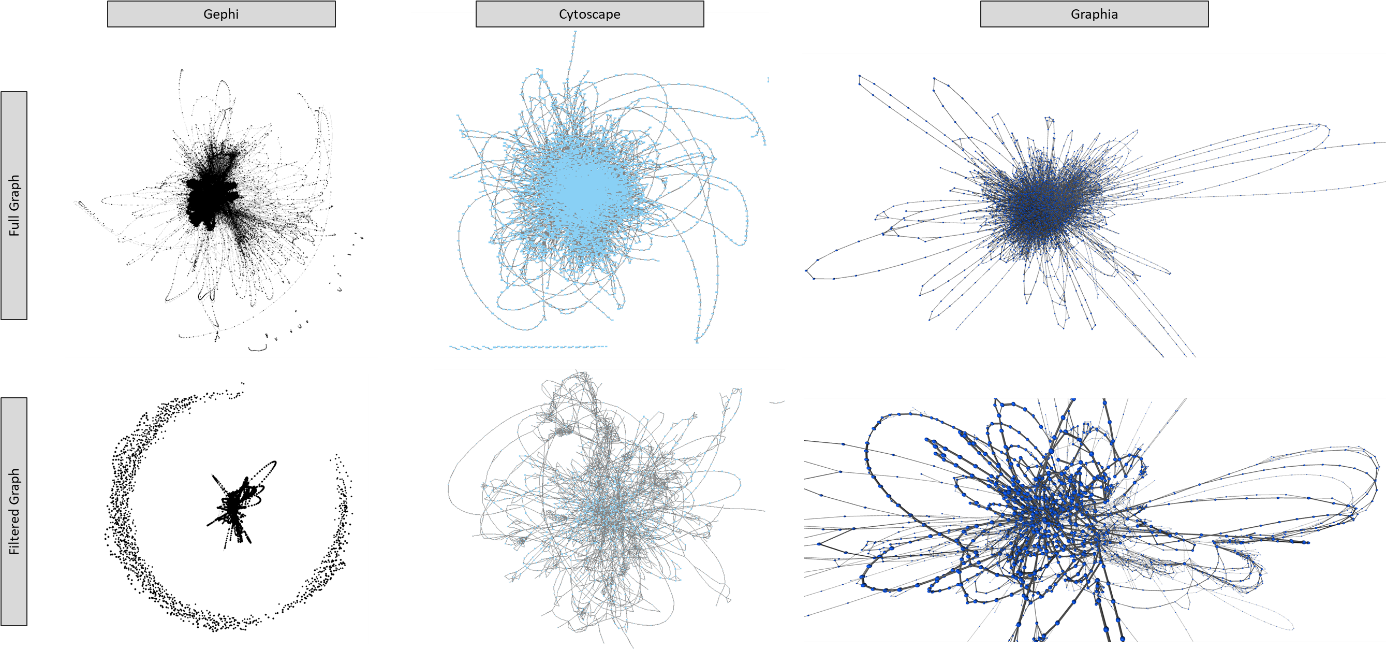


**Supplementary Figure 1 | Comparison of the layout of pangenome synteny networks in Gephi, Cytoscape and Graphia.** The same “full” pangenome synteny network (all nodes and edges; 7,228 nodes and 34,500 edges) and a filtered subset (only nodes in >10 and <770 genomes; 2,711 nodes and 4,110 edges) were loaded to each tool, in the .graphml file format. Graphia layouts are shown in 3D.


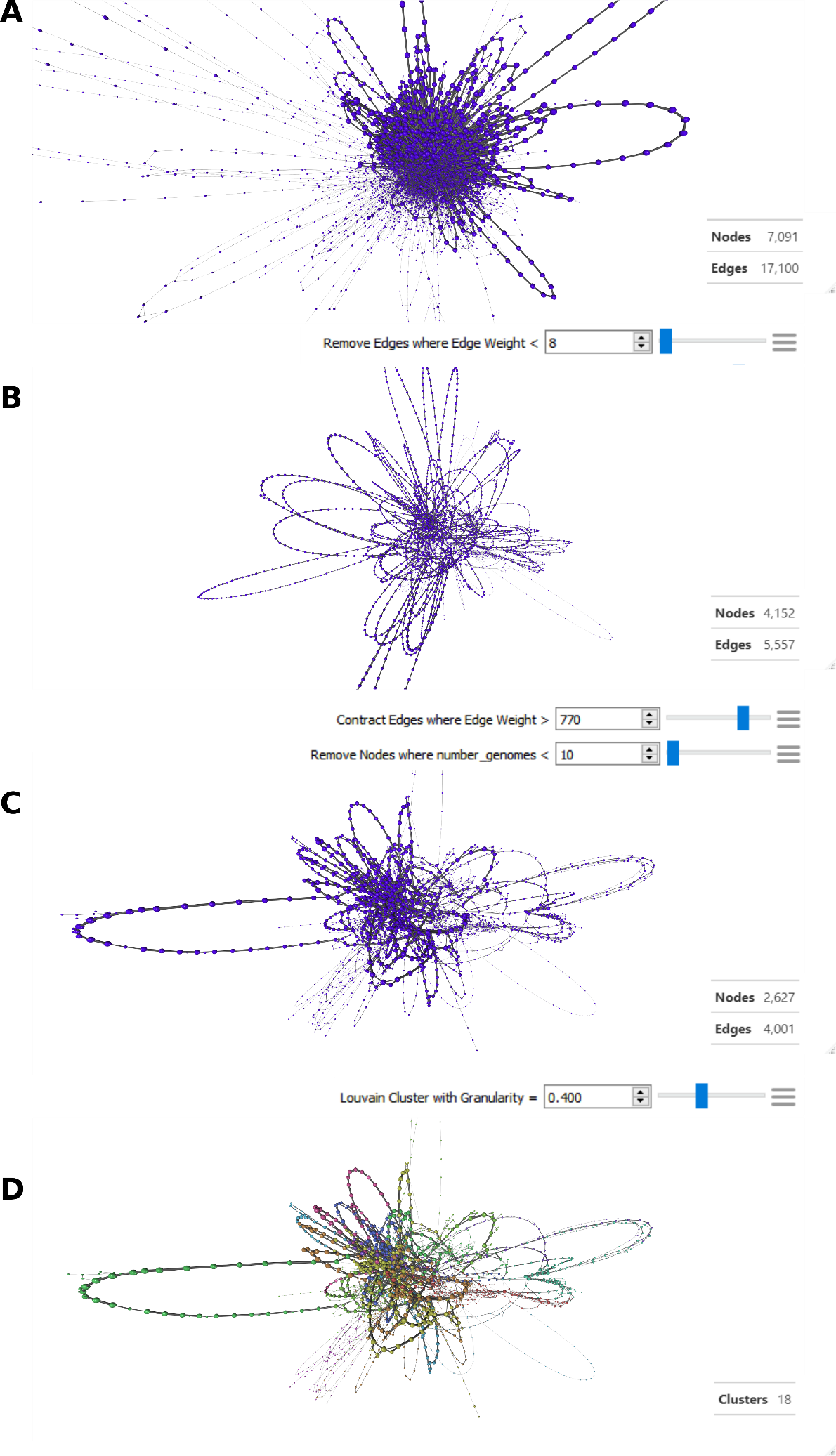


**Supplementary Figure 2.** **Simplification of pangenome synteny graphs increases visual clarity and usability.** In each panel, nodes represent genes and edges represent the syntenic arrangement of genes (occurrence next to one another in genomes). Nodes and edges are sized according to weight (the larger the node or thicker the edge, the more genomes in which that gene or relationship occur). Network node/edge counts are shown at each step to demonstrate simplification of the graph. **(A)** Full synteny graph, visualising the network file of the *S. aureus* pangenome on first import into Graphia. **(B)** Network after removal of low weight (*n* <8) edges. **(C)** High edge weights contracted, collapsing stretches of core gene nodes into single “multi-element” nodes. **(D)** Network clustered using Louvain clustering algorithm (LCi = 0.400), resulting in 18 clusters. Nodes coloured by LC.
